# Genomic Responses to Arsenic in the Cyanobacterium *Synechocystis* sp. PCC 6803

**DOI:** 10.1371/journal.pone.0096826

**Published:** 2014-05-05

**Authors:** Ana María Sánchez-Riego, Luis López-Maury, Francisco Javier Florencio

**Affiliations:** Instituto de Bioquímica Vegetal y Fotosíntesis, Universidad de Sevilla-CSIC, Sevilla, Spain; University of Freiburg, Germany

## Abstract

Arsenic is a ubiquitous contaminant and a toxic metalloid which presents two main redox states in nature: arsenite [As^III^] and arsenate [As^V^]. Arsenic resistance in *Synechocystis* sp. strain PCC 6803 is mediated by the *arsBHC* operon and two additional arsenate reductases encoded by the *arsI1* and *arsI2* genes. Here we describe the genome-wide responses to the presence of arsenate and arsenite in wild type and mutants in the arsenic resistance system. Both forms of arsenic produced similar responses in the wild type strain, including induction of several stress related genes and repression of energy generation processes. These responses were transient in the wild type strain but maintained in time in an *arsB* mutant strain, which lacks the arsenite transporter. In contrast, the responses observed in a strain lacking all arsenate reductases were somewhat different and included lower induction of genes involved in metal homeostasis and Fe-S cluster biogenesis, suggesting that these two processes are targeted by arsenite in the wild type strain. Finally, analysis of the *arsR* mutant strain revealed that ArsR seems to only control 5 genes in the genome. Furthermore, the arsR mutant strain exhibited hypersentivity to nickel, copper and cadmium and this phenotype was suppressed by mutation in *arsB* but not in *arsC* gene suggesting that overexpression of *arsB* is detrimental in the presence of these metals in the media.

## Introduction

Arsenic is an ubiquitous toxic metalloid and a human carcinogen that causes serious health problems in many places of the world where arsenic contents in drinking water are well over the recommended limits [1]. Arsenic is present in two biologically active forms, arsenate [As^V^] and arsenite [As^III^], depending of the redox potential of the environment. Arsenate is a phosphate analog, enters the cells through phosphate transporters and its toxicity is mediated by replacing phosphate in essential biochemical reactions such as oxidative phosphorylation and glycolysis [2], [3], [4], [5]. The resulting arseno-compounds are extremely labile and hydrolyze spontaneously at milliseconds rates making them unable to be used by living organisms [2], [3], [4], [5], [6]. On the other hand, arsenite enters the cell through aquaglyceroporins and exerts its toxicity through binding to dithiols, forming arsenothiols that perturb protein function and that ultimately generate reactive oxygen species (ROS) [7], [8], [9], [10]. Because of the high affinity for sulfur, arsenite is able to bind to the main redox buffer in the cells, glutathione (GSH) forming As^III^-GSH_2_ and depletes its pool [11], thus contributing to ROS generation. Furthermore, phytochelatins, which are GSH polymers (n = 2–16), are well known to contribute to arsenite resistance [12]. Recently, it has been shown that arsenite is able to inhibit protein folding and induce protein aggregates interfering with normal cell function [13], [14], [15]. Despite being toxic, arsenic is also used by some microorganisms as electron acceptor in an anaerobic respiratory chain, electron donor to grow chemo-lithotrophically, and even for anoxigenic photosynthesis [16], [17]. Furthermore, it has been postulated that arsenic played an important role during early life on earth before appearance of oxygen [18], [19].

Because of the wide use and distribution of arsenic compounds, arsenic resistance is wide spread among living organisms. Many resistance systems consist in reduction of arsenate to arsenite followed by export of the latter outside the cell or its transport to the vacuole. Arsenate reduction to arsenite is catalyzed by arsenate reductase, an enzymatic activity carried out by at least three non-related families. These reductases use the thioredoxin, glutaredoxin or mycoredoxin systems as electron donors [20], [21], [22], [23], [24], [25], [26]. Arsenite export is mediated by two families of proteins: ArsB proteins, that are present only in bacteria [27], and Acr3 proteins, which are more widely distributed in different organisms including bacteria, fungi and plants [28], [29]). Another detoxification system that is present from bacteria to animals is arsenic methylation, which conjugates arsenic to methyl groups and can lead to formation of arsenic volatile species [30], [31]. Recently, it has been shown that bacteria are able to survive high arsenate concentrations by a mechanism involving rRNA degradation and selection of a subpopulation that is resistant to arsenate. This mechanism seems to account for the growth of the highly resistant Halomonas GFAJ-1 in media containing arsenate and lacking phosphate [32], [33].

In cyanobacteria arsenic metabolism and resistance is best understood in the model cyanobacterium *Synechocystis* sp. PCC 6803 (hereafter referred to simply as *Synechocystis*). The main arsenic resistance mechanism is mediated by an operon of three genes (*arsBHC*) that is regulated by an unlinked *arsR* homolog [24]. The operon includes an Acr3 arsenite transporter gene, *arsB*, an *arsH* homolog that codes for a FMN-quinone reductase without a clear function in arsenic resistance [24], [34], and an arsenate reductase gene, *arsC*, which codes for a new type of hybrid arsenate reductase that although related to thioredoxin dependent ones, use the glutathione/glutaredoxin system for reduction [21], [22], [23]. At least two other resistance determinants have been described in *Synechocystis*: an additional arsenate reductase from the *E. coli* family (encoded by two nearly identical genes *arsI1* and *arsI2* genes; [23]) and an arsenite methylase gene *arsM* [35]. ArsI is only essential for arsenic resistance in the absence of ArsC, probably due to its low level of expression [23]. The role of ArsM in resistance has not been tested *in vivo*, but *E. coli* strains carrying *arsM* genes from different cyanobacteria are more resistant to arsenite. Furthermore, ArsM is able to methylate arsenite to the volatile trimethylarsine [TMA(III)] using S-adenosyl methionine and glutathione as methyl donors *in vitro* [35].

Here we describe the global genomic responses to both arsenate and arsenite in wild type (WT) *Synechocystis* and mutants affected in the arsenic resistance system. Both treatments induced a very similar response in WT cells, probably due to the highly efficient arsenate reduction mechanism that converts all arsenate into arsenite. Nevertheless, analysis of the responses on a SARS12 strain (that lacks all arsenate reductases) showed a differential regulation of genes involved in metal homeostasis, Fe-S cluster biogenesis and anabolic pathways. The SARSB strain (that lacks the *arsB* arsenite transporter) showed a response similar to the WT strain after 1h. However, this response was sustained in time in the SARSB strain, and transient in the WT strain, and probably reflects the inability of the SARSB strain to detoxify arsenite. On the other hand, the mutant strain lacking the *arsR* gene (the SARSR strain), which expresses the *arsBHC* operon at high levels, did not respond to arsenite and was resistant to both arsenite and arsenate. Surprisingly the SARSR strain also presented sensitivity to nickel, cadmium and copper in the media. This sensitivity depends on the presence of the *arsB* gene, suggesting that overexpression of ArsB leads to altered permeability to metals.

## Results and Discussion

### Responses to arsenate and arsenite are highly correlated in wild type *Synechocystis*

**Figure 1:**
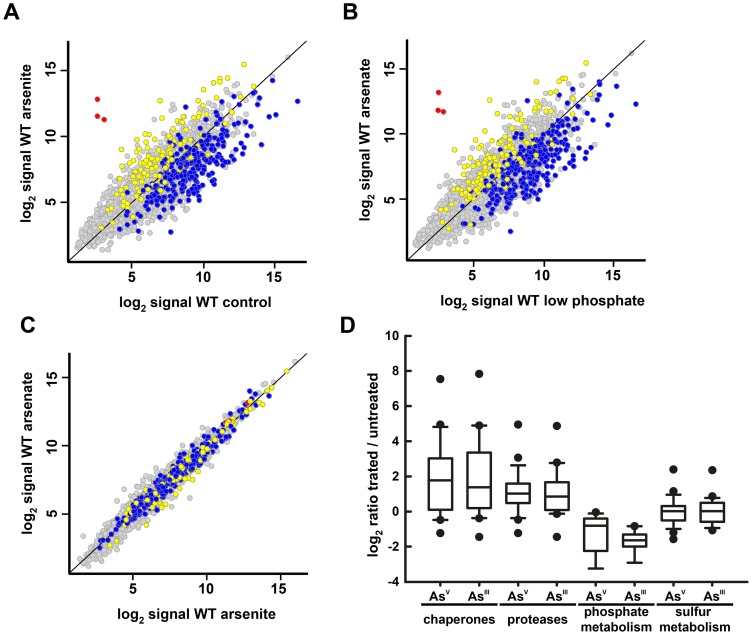
Global responses to arsenic in wild type *Synechocystis* cells. A. Scatter plot showing comparison between expression profiles of WT cells treated with 1(y axis) and untreated cells (x axis). Data represent the average signal of four independent hybridizations. CTR (Core Transcriptional Response) up-regulated genes are colored in yellow, CTR down-regulated genes in blue and the *arsBHC* operon in red. B. Scatter plot showing comparison between expression profiles of WT cells grown in low phosphate media treated with 50 mM arsenate for 1 h (y axis) and untreated cells grown in low phosphate media (x axis). C. Scatter plot showing comparison between expression profiles of WT cells grown in low phosphate media treated with 50 mM arsenate for 1 h (y axis) and cells grown in BG11C treated with 1 mM arsenite for 1 h (x axis). D. Box plot showing ratios of treated *vs.* untreated cells of the categories cited in the main text.

In order to characterize the physiological responses to the presence of arsenic in *Synechocystis* we have performed genome-wide analysis of gene expression in response to both arsenite and arsenate. WT cells were exposed to 1 mM arsenite or 50 mM arsenate for 1 h, RNA was extracted and hybridized to Agilent 15K one-color arrays; control samples from untreated cells were also used for microarray hybridization. These concentrations and sampling times were chosen as they produced growth inhibition without compromising cell viability and showed maximal induction for the *arsBHC* operon [23], [24]. Four independent biological replicates were performed and Limma was used (2.5 fold-change and *P<*0.01) in order to identify differentially expressed genes. For the arsenate treatment cells were grown in low phosphate media before arsenate addition (containing only 15% of the normal phosphate which is ∼30 µM) in order to detect growth inhibition by arsenate in the WT strain [23], [24]. Hence, differential expression between BG11C (which is phosphate-replete and contains 200 µM of phosphate) and low phosphate BG11C (30 µM phosphate) was also analyzed. Under this experimental conditions there were no genes differentially expressed (Figure S1 in File S1). In contrast, addition of arsenite significantly changed expression of 421 genes (179 induced, 242 repressed; Figure 1A; Table S1) whereas arsenate changed expression of 580 genes (266 induced; 314 repressed; Figure 1B; Table S1) when compared to control conditions. Although the number of genes was slightly different the responses were highly correlated (Figure 1C; R^2^ = 0.98). These highly correlated responses are probably due to the presence of a highly effective arsenate reduction mechanism in *Synechocystis*, which has three arsenate reductase genes. Two of these genes (*arsI1* and *arsI2*) are expressed constitutively while the third one (*arsC*) is rapidly induced after arsenate addition and the combined action of these three enzymes likely leads to a quick reduction of intracellular arsenate to arsenite [23], [24]. In order to further identify cellular processes that changed after the arsenic treatments, gene set enrichment analysis was performed using gene lists extracted from cyanobase functional categories, GO terms and literature hand-curated gene lists using GSEA (see material and methods and supplementary materials). In agreement with the data presented above both treatments significantly induced or repressed almost identical gene sets (Table S2).

#### Stress response

The main response to both treatments was the induction of a general stress response that included repression of growth related genes, such as translation related processes (see Tables S1 and S2) or energy generation systems (Photosynthesis, ATP synthetase and Respiration; Table S2), and induction of genes that are also up-regulated in other stresses which includes functional categories such as chaperones, drug and analog sensitivity and protein degradation (Table S2). Regulation of growth or stress related groups of genes have been described as part of the Core Transcriptional Response (CTR), because they are usually regulated in response to different stress conditions [36]. As shown in Figure 1A and B CTR genes (colored in yellow for genes up-regulated in most conditions and in blue for genes down-regulated) are away from the diagonal showing their change in expression after both treatments. This response has been proposed to be controlled by several regulatory genes including Hik33, Hik34, PerR, Rre1, Rre31 and SigB [36]. *sigB*, *hik34* and *perR* genes were all induced by the arsenic treatments (Table S1), suggesting that the regulatory network acting in response to a general stress was activated.

Chaperones and proteases constituted one group of induced genes (Figure 1D; Tables S1 and S2), with only a few genes from this list not induced. Although some of the chaperone genes like *dnaK2*, *htpG* or *hspA* have been described to be up-regulated after several stress conditions [37], [38], [39], [40], [41], [42], a general induction of this group has not been previously described. Recently, it has been shown that arsenite is able to inhibit nascent protein folding both *in vitro* and *in vivo* in yeast [13], [14], [15] and Archea [15]. These results suggest that in *Synechocystis* arsenite is also able to cause protein damage and that chaperones and proteases are induced in order to repair or degrade these damaged proteins. In fact, a similar response has been observed in yeast and a bacterium, in which arsenite induces expression of chaperones and the proteosome in order to refold and/or degrade protein aggregates [43], [44], [45], [46]. Moreover, yeast mutants defective in the chaperonin TRiC complex or proteosome regulation are more sensitive to arsenite, suggesting that refolding and degradation of misfolded proteins are essential for survival after arsenite stress [15], [43], [44].

**Figure 2:**
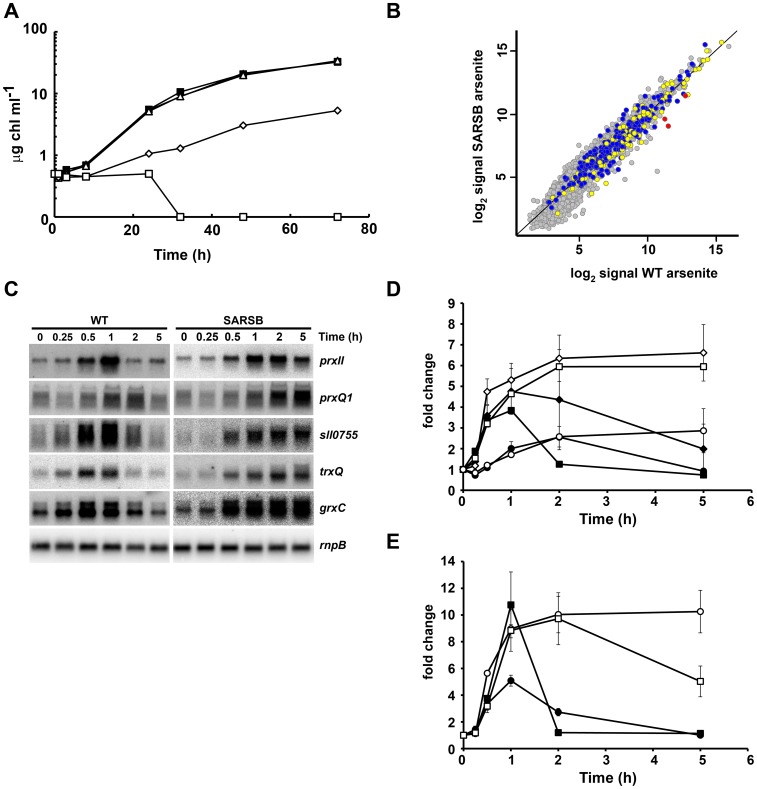
SARSB strain shows a sustained stress response. A. Growth of WT and SARSB strains in different arsenite concentrations. Exponentially growing cells of WT (filled symbols) and SARSB (open symbols) were diluted to 0.5 µg chl ml^−1^ in media containing 100 µM arsenite (squares), 50 µM arsenite (diamonds) or without added arsenite (triangles). Growth was monitored following chlorophyll concentration. B. Scatter plot showing comparison between expression profiles of SARSB (y axis) and WT (x axis) treated with 1 mM arsenite for 1 h. Data represents the average signal of two hybridizations for SARSB and four hybridizations for WT. CTR up-regulated genes are colored in yellow, CTR down-regulated genes in blue and the *arsBHC* operon in red. C. Northen blot analysis of *prxII*, *prxQ1*, *2cys-prx*, *trxQ* and *grxC* expression in response to 1 mM arsenite treatment in WT and SARSB strains. Total RNA was isolated from WT or SARSB cells grown in BG11C medium after addition of arsenite 1 mM. Samples were taken at the indicated times. The filters were subsequently hybridized with *prxII*, *prxQ1*, *2cys-prx*, *trxQ, grxC* and *rnpB* probes. D. Quantification of relative mRNA levels of *prxQ1* (circles), *trxQ* (squares) and *grxC* (diamonds) in response to 1 mM arsenite treatment in the WT (filled symbols) and the SARSB (open symbols) strains. Radioactive signals of three independent experiments for each strain were quantified and averaged. RNA levels were normalized with the *rnpB* signal in all strains. Plots of relative mRNA levels *vs.*time were drawn; error bars represent SE. E. Quantification of relative mRNA levels of *prxII* (squares) and *2cys-prx* (circles) in response to 1 mM arsenite treatment in the WT (filled symbols) and SARSB (open symbols) strains. Radioactive signals of three independent experiments for each strain were quantified and averaged. RNA levels were normalized with the *rnpB* signal in all strains. Plots of relative mRNA levels *vs.* time were drawn; error bars represent SE.

Induction of several genes coding for proteins involved in redox scavenging like *trxQ* (*slr0233*), *2cys*-*prx* (*sll0755*), *prxII* (*sll1621*), *gpx1* (*slr1171*) and *sodB* (*slr1516*) was observed. In addition several gene lists extracted from microarray experiments interrogating oxidative stress were also enriched in the GSEA analysis (Tables S1 and S2; Figure 2C–E). This supports that arsenic (probably arsenite) generates ROS and that CTR regulation could be mediated by ROS [36]. Furthermore, in *Pseudomonas* and yeast, several mutants affected in oxidative stress responses or its regulation are sensitive to arsenite [47], [48], [49], [50], [51]. This data strongly suggests that the oxidative stress response is essential for survival under arsenic stress. Moreover, a similar response was also observed in *Synechocystis* after cadmium addition, suggesting that both non-biologically active metals have a similar effect on cells [52]. This is further supported by the enrichment of cadmium induced gene lists in the GSEA analysis (Table S2). Both arsenic and cadmium share a strong affinity for thiol binding and this could explain why they have similar effects in cells as they would interfere with similar set of proteins.

#### Arsenic related genes

The most highly induced genes in the genome after both arsenate or arsenite addition were the *arsBHC* operon, with *arsB* being induced 1548±196 and 1226±357, *arsH* 676±240 and 521±173 and *arsC* 447±110 and 318±97 times for arsenate and arsenite, respectively. These values are consistent with the strong polarity previously described by us for this operon; [24]. Unfortunately, *arsI* probes were not included in our array design and therefore we analyzed its expression by qRT-PCR. *arsI* (it is not possible to distinguish between them as they are 99% identical) was not induced by arsenate or arsenite, was expressed at low levels and not regulated by ArsR ([23] and Figure S2 and S7 in File S1). The expression profiles of the three arsenate reductase genes correlate with their roles in arsenic detoxification *in vivo*, as mutants in *arsC* are much more sensitive to arsenic than mutants in *arsI* [23]. In contrast to what has been described, the *arsM* (*slr0303*) gene was not induced by treatments with arsenite or arsenate in any of the strains analyzed, perhaps because growth conditions and incubation times were completely different to those reported previously [35]. Finally, the effects of mutation in *arsM* have not been analyzed *in vivo*, but it is unlikely that it will significantly contribute to arsenic resistance in short term treatments because accumulation of methylated species of arsenic occurred only after long periods of arsenic exposure [35]. This suggests that arsenic methylation could be a mechanism of adaptation to chronic exposure to arsenic.

Phosphate has a prominent role in protecting cells from arsenic toxicity, especially from arsenate, which enters the cells through phosphate transporters and acts as a phosphate analog [2], [53], [54], [55], [56]. In fact, in *Synechocystis* a reduction in phosphate concentration in the media is essential to detect growth inhibition by addition of arsenate, even in mutants affected in arsenate resistance [23], [24]. Phosphate transport genes were repressed by both arsenite and arsenate (Figure 1D and Figure S2 in File S1; Tables S1 and S2), suggesting that cells responded to arsenic preventing further acquisition of arsenate after both treatments. In other bacteria the low affinity phosphate transport system is repressed by arsenate but the high affinity transport system is induced in several bacteria [45], [46], [57], [58]. This pattern of expression prevents incorporation of arsenate into the cells because the high affinity systems generally have a better discrimination towards phosphate [54]. *Synechocystis* (and most cyanobacteria) present two high affinity transport systems for phosphate (pst-1 and pst-2) but no low affinity transport system [59]. The pst-1 system (that includes *sphXpstS1C1A1B1B1′*operon) is expressed at higher levels than the pst-2 system (*pstS2C2A2B2* operon) under phosphate repleted conditions, although both of them are induced in response to phosphate starvation [59]. The absence of low affinity phosphate transport system and the presence of two high affinity transport systems could explain the high resistance to arsenate in *Synechocystis*, a situation that is similar to the recently described highly arsenate resistant *Halomonas* GAFJ-1, which also presents two high affinity phosphate transport systems [54], [60]. In *Halomonas* GAFJ-1 the genes coding for phosphate transport systems are induced by phosphate starvation but its expression is not affected by arsenate [54]. The specificity of *Synechocystis*' transporters has not been tested *in vitro*, but *in vivo* more than 3000 fold difference in arsenate over phosphate concentration is needed to observe a negative effect in growth [23], [24], suggesting that the specificity for phosphate could be in this range. Repression of phosphate transport genes in *Synechocystis* is not simply a consequence of the activation of a general stress response since these genes are induced by other stress treatments such as high light and nitrogen starvation [59].

Another protein that has been shown to be involved in arsenic resistance in other organisms is aquaporin. Aquaporins mediate non specific uptake of arsenite and mutants in the corresponding genes are usually arsenite resistant [7], [8], [9], [10], [61]. The only gene coding for this protein in *Synechocystis* (*aqpZ; slr2057*) was repressed after both arsenate and arsenite (10 fold in arsenate and 6 fold in arsenite; Table S1). This repression would probably minimize arsenite uptake. A similar response is also observed in plants treated with either arsenate or arsenite in which some of the aquagliceroporins genes from the Nod26-like (NIP subfamily) subfamily, are repressed. This family of transporters has been suggested to be responsible for arsenite transport in roots [62], [63], [64].

#### Sulfur and glutathione metabolism genes

Sulfur metabolism genes have been shown to be induced after arsenic treatments in bacteria [58], [65], yeast [44], [51], [66] and plants [63], [67], [68]. These genes include sulfate transport, sulfur assimilation and cysteine synthesis genes, which are needed in order to increase GSH contents. GSH is essential for arsenic resistance in many organisms, although several mechanisms have been described. In *E. coli*, yeast, plants and *Synechocystis* GSH is essential for arsenate reductase activity [20], [21], [23], [69], [70], [71], [72], [73]. In budding yeast GSH is also conjugated to arsenite and this complex is sequestered in the vacuole, while in fission yeast and plants GSH is used to synthesize phytochelatin (PC) which binds arsenite and prevents its toxicity [12], [62]. In all cases GSH pools are reduced as a consequence of arsenic treatments therefore contributing to generate oxidative stress. In *Anabaena* sp. PCC 7120, a filamentous cyanobacterium that synthesizes PC, both *pcs* (the gene coding for PC syntase) mRNA and PC contents increases in response to arsenate and glutathione reductase activity is also induced [74]. In contrast, genes related to sulfur metabolism did not change its expression (Figure 1D) after arsenate or arsenite treatments in *Synechocystis*, even though GSH is essential for arsenate reduction and resistance [21], [22], [23]. Furthermore, *Synechocystis* genome lacks a gene coding for a canonical GSH reductase, and no NADPH-dependent GSH reductase activity is detected in crude extracts ([75], [76] and our unpublished observations).

#### Metal metabolism genes

Besides arsenic resistance genes, several other genes related to metal metabolism were also induced by both arsenate and arsenite treatments. Most of these genes are clustered together in *Synechocystis*' genome (Tables S1 and S2). These genes are involved in Ni, Zn, Co and Cu resistance [77], [78], [79], [80], [81]. Interestingly, only the genes under the control of intracellular metal sensors (*nrsD*, *ziaT*, *corT*) were induced [77], [78], [79], [80]. This data suggests that arsenic (probably arsenite) can bind to the metal binding sites of these cytosolic metal sensors and affect their activity. In agreement with these, the genes coding for the two copper importing ATPases (*pacS* and *ctaA*) and the copper chaperone (*atx1*) were also induced (Table S1). The regulatory circuits for these three copper-related genes are unknown but they are expected to be under the control of an intracellular metalloregulator. This protein should be able to detect internal copper levels and therefore should be susceptible to be bound by arsenic (arsenite).

### SARSB shows a sustained response to arsenite

The expression profiles of the *arsB* mutant (the SARSB strain) that is sensitive to both arsenate and arsenite [24] was also analysed in both untreated and treated conditions. In untreated conditions, the *arsBHC* operon was expressed at higher levels in the SARSB than in the WT strain (Figure S3 in File S1), probably due to the constitutive promoter present in the CK1 cassette used for the *arsB* disruption, but no other genes were differentially expressed. Furthermore, the gene expression profile of the SARSB strain after the treatment with 1 mM arsenite, a concentration that completely inhibits growth of this strain (Figure 2A, Figure S4A in File S1 and [24]), was very similar to the gene expression profile of the WT strain (Figure 2B, R = 0.93). The only differentially expressed genes were the *arsBHC* operon, which under this condition was expressed at lower levels in the SARSB strain than in the WT strain. This is also probably due to the CK1 cassette used for SARSB construction, which interferes with transcription from the *arsBHC* promoter. In order to further analyze the expression profile of this strain, expression of several redox genes was analyzed after arsenite treatment for 24 h both in the WT and the SARSB strains. Thioredoxins and glutaredoxins genes (especially *trxQ* and *grxC*) were induced transiently in the WT strain, while this induction was maintained in the SARSB strain for at least 24 h (Figure 2C and 2D; Figure S5 in File S1). Similar effects were observed when peroxiredoxin genes were analyzed. Out of the 5 peroxiredoxin genes present in *Synechocystis*, only *sll0755* (*2cys-prx*), *slr0242* (*prxQ1*) and *sll1621* (*prxII*) were transiently induced by arsenite in the WT strain, but again their expression was maintained in the SARSB strain (Figure 2C and 2E and Figure S5 in File S1). This data suggests that arsenite alters the redox state of the cells causing oxidative stress in WT cells. This alteration of the redox state is transient in WT cells, which return to basal levels after 2 h, while is maintained in the SARSB strain. This strain lacks ArsB to export arsenite, therefore arsenite accumulation probably leads to induction of oxidative stress. The mechanism by which arsenite generates oxidative stress is not clear, but it has been proposed that arsenite binds dithiols (inhibiting many redox reactive proteins such as thioredoxins or glutaredoxins), it depletes GSH pools and inhibits protein folding. All these mechanisms will ultimately generate ROS and therefore activate an oxidative stress response as observed in our results (Figure 1). In fact, induction of the redox genes was only noticed after 30 min (Figure 2C), while these genes are induced earlier after H_2_O_2_ or high light treatments [40], [42], [82], [83]. In contrast, the *arsBHC* operon was already induced at earlier times [24], suggesting that arsenite causes an oxidative stress only when it accumulates in the cell. The sustained oxidative stress response in the SARSB strain indicates that this strain accumulates arsenite, causing a permanent damage that ultimately kills the cells.

### SARS12 shows a different expression profile in response to arsenate

**Figure 3:**
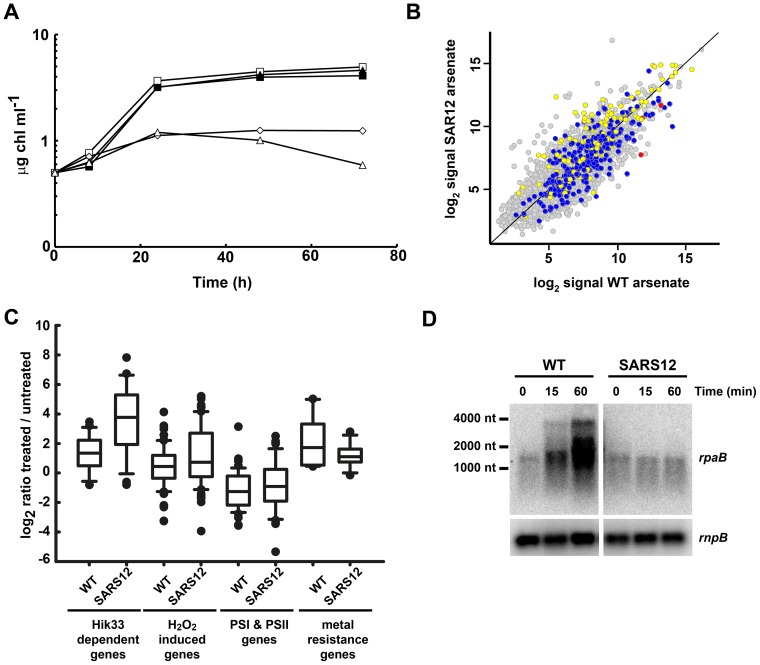
The SARS12 strain shows a different response to the WT strain. A. Growth of WT and SARS12 strains in the presence of different arsenate concentrations. Exponentially growing cells of WT (filled symbols) and SARS12 (open symbols) were diluted to 0.5 µg chl ml^−1^ in low phosphate media containing 100 mM arsenate (triangles), 50 mM arsenate (diamonds) or without added arsenate (squares). Growth was monitored following increase in chlorophyll concentration. B. Scatter plot showing comparison between expression profiles of SARS12 (y axis) and WT (x axis) treated with 50 mM arsenate for 1 h. Data represent the average signal of two hybridizations for SARS12 and four hybridizations for WT. CTR up-regulated genes are colored in yellow, CTR down-regulated genes in blue and the *arsBHC* operon in red. C. Box plot showing ratios of treated *vs.* untreated of the categories cited in the main text in the WT and SARS12 strains. D. Northen blot analysis of *rpaB* expression in response to 50 mM arsenate treatment in the WT and SARS12 strains. Total RNA was isolated from WT or SARS12 cells grown in low phosphate medium after addition of 50 mM arsenate. Samples were taken at the indicated times. The filters were hybridized with *rpaB* and subsequently stripped and re-hybridized with an *rnpB* gene probe as a control.

In order to study the specific effect of arsenate in gene expression, a strain lacking all the arsenate reductases genes (SARS12 strain) was used. This strain was treated with 50 mM arsenate and its expression profile was compared to the WT treated with 50 mM arsenate. Under control conditions there were no genes differentially expressed between SARS12 and WT cells other than *arsC* (Table S3; Figure S3 in File S1). In contrast to what was observed in the SARSB strain treated with arsenite, the profile of the SARS12 strain treated with arsenate was much more different from the WT profile (Figure 3B; Tables S3 and S4). Despite the fact that the SARS12 strain is highly sensitive to arsenate and lacks all known enzymatic arsenate reduction mechanisms (Figure 3A, Figure S4 in File S1 and [24]), the *arsBHC* operon (400 fold for *arsB*) was induced in response to arsenate in the SARS12 strain (Table S3). The *arsBHC* operon is repressed by ArsR, which only respond to reduced forms of metalloids [24]. Therefore, this suggests that arsenate could be reduced *in vivo* by a system independent of ArsC and ArsI (which are absent in SARS12). In Arabidopsis and human cell lines a similar effect has been also observed and it was proposed that arsenate could be reduced non-enzymatically by mono or dithiols or unspecifically by other enzymes [71], [84]. Alternatively, traces of arsenite could be contained in the arsenate salt, which was used at high concentration (50 mM final concentration) and therefore even small arsenite contamination could be enough to induce *arsBHC* expression.

GSEA analysis in this mutant identified several groups of genes that were differentially expressed between WT and SARS12 strains treated with arsenate (Table S4). Several of the lists extracted from the literature related to oxidative stress were enriched in SARS12 when compared with WT. These include genes induced by H_2_O_2_, and those controlled by Hik34 and Hik33 histidine kinases [83], suggesting that the stress response was activated at higher levels than in WT under this condition (Figure 3C). Despite this, many of the energy generation related gene lists (PSI, PSII, PBS or ATP synthase), which are repressed after many stress treatment including arsenic treatment, were not differentially expressed (Figure 3C) in the SARS12 strain.

One group of genes that was enriched in the SARS12 strain was high light induced genes (Table S4), which included *hliA*, *hliB*, *hliC*, *isiA* and *nblA*. All these genes are repressed by RpaB [85], [86], the response regulator coded by the gene downstream of *arsC.* This raised the possibility that *rpaB* mRNA levels could be affected in the SARS12 strain causing the up-regulation these genes. In fact *rpaB* expression levels were lower in the SARS12 strain than in the WT strain in the microarray analysis (induction is 4.3 in SARS12 vs. 16.48 in WT). In order to confirm these results *rpaB* expression was analyzed in the WT an SARS12 strains by northern blot and RT-PCR. In the WT strain *rpaB* was co-transcribed with *slr0948* in untreated samples and together with *arsBHC* (and *slr0948*) in response to arsenite (Figure 3D and Figures S2 and S6 in File S1). In contrast, in the SARS12 strain *rpaB* was not induced by arsenite and it was not co-transcribed with the *arsBHC* operon (Figure 3D). This lower level could be caused because the insertion of the C.K1 cassette in *arsC* could have a polar effect in *rpaB* transcription. In *Synechococcus* sp. PCC 7942 RpaB activity is negatively regulated by phosphorylation by NblS, which is the homolog of Hik33. As Hik33 controlled genes were enriched (induced) in the SARS12 strain when compared to WT in the GSEA analysis (see above), this suggests that Hik33-RpaB signaling pathway was suppressed after arsenate addition. Even more, *sigD* which is under the control of the Hik33-RpaA (Rre31) [83], [87], was also induced at much higher level in the SARS12 strain (23 fold) than in the WT (1.7 and 5 fold in arsenate or arsenite, respectively). The genes under SigD control have not been identified in a genome-wide scale, although some of the genes that are induced after high light (*hli* genes, *psbA2* and *psbA3*) treatment were less induced in a mutant lacking *sigD* [88], [89], [90]. These genes were also induced at higher levels in SARS12 (see above) reinforcing the idea that SigD was activated after arsenate treatment in SARS12. Furthermore, *rpoD3* (the homolog of *sigD*) is repressed by RpaB in *Synechococcus* sp PCC 7942 [91], [92] suggesting that regulation of *sigD* gene could be also mediated by RpaB in *Synechocystis*.

Another group of genes that were repressed in SARS12 when compared to WT were metal transport genes. This group of genes includes Ni, Co, Zn and Cu resistance systems, which were induced after both arsenate and arsenite treatments in WT. Of these only those regulated by cytosolic metalloregulators were induced in WT (less induced in SARS12) suggesting (Figure 3C) that arsenic affects intracellular metal sensing. In this regard, the effect observed in WT cells after both treatments was most probably mediated by arsenite, which is expected to be accumulated at much lower levels in SARS12 than in WT cells, explaining the higher induction of these genes in WT. Finally, genes involved in Fe-S cluster assembly were induced at lower levels in SARS12 than in WT, despite a higher induction of oxidative stress response in the mutant (Figure 3C). These results suggest that arsenite could target these cofactors directly *in vivo* and that the reduced amounts of arsenite accumulated in the mutant partially prevent the damage to Fe-S clusters.

### ArsR controls expression of *arsBHCrpaBslr0948* operon and *sll0914*

**Figure 4:**
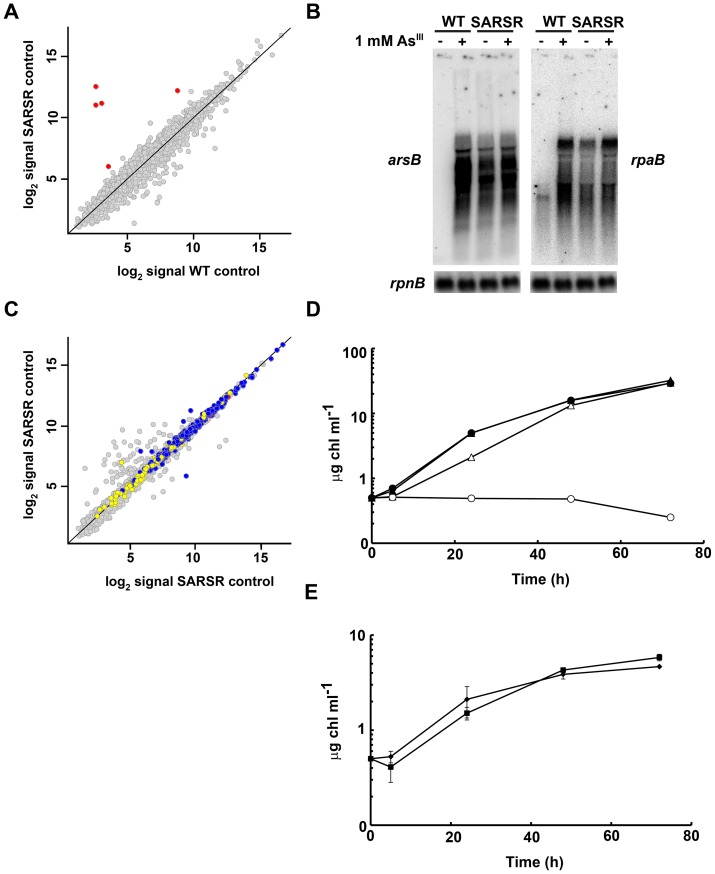
ArsR only controls expression of *sll0914* and *arsBHCrpaBsll0947* operon. A. Scatter plot showing comparison between expression profiles of SARSR (y axis) and WT (x axis) in untreated samples. Data represents the average signal of two hybridizations for SARSR and four hybridizations for WT. In red are colored genes that are differentially regulated in the SARSR strain. B. Northern blot analysis of *arsB* and *rpaB* expression in response to 1 mM arsenite treatment in the WT and SARSR strains. Total RNA was isolated from WT or SARSR cells grown in BG11C at the indicated times after arsenite addition. The filters were subsequently hybridized with *arsB* and *rpaB* and *rnpB* probes. C. Scatter plot showing comparison between expression profiles of the SARSR strain treated with 1 mM arsenite for 1 h (y axis) and untreated (x axis). Data represents the average signal of two hybridizations. CTR up-regulated genes are colored in yellow, CTR down-regulated genes in blue and the *arsBHC* operon in red. D. Growth of the WT and SARSR strains in the presence of different arsenite concentrations. Exponentially growing cells of WT (filled symbols) and SARSR (open symbols) were diluted to 0.5 µg chl ml^−1^ in BG11C containing 3 mM arsenite (triangles), 5 mM of arsenite (circles) or without added arsenate (squares). Growth was monitored following increase in chlorophyll concentration. E. Growth of the WT and SARSR strains in the presence of arsenate. Exponentially growing cells of the WT (diamonds) and SARSR (squares) strains were diluted to 0.5 µg chl ml^−1^ in low phosphate media containing 100 mM of arsenate (. Growth was monitored following chlorophyll concentration; data represent average of 3 independent experiments and error bars represent SE.

We have previously reported that *arsBHC* operon is regulated by ArsR which is encoded by an unlinked gene [24]. In order to identify genes regulated by ArsR we have performed expression profiling of SARSR in both control conditions and after arsenite addition. Statistical analysis identified only 5 genes that were differentially expressed in SARSR *vs.* WT control cells. These included the *arsBHC* operon, as expected, but also the gene that is downstream to it, *rpaB*, and the gene that is upstream of *arsB* but in the opposite strand, *sll0914* (Figure 4A; Table S5). *rpaB* was expressed as a dicistronic mRNA (∼1600 pb) together with *slr0948* (the gene located downstream to *rpaB* and co-transcribed also when analysed by RT-PCR; see Figures 3D, 4B and Figure S6 in File S1) in control cells. In contrast, it was expressed as a polycistronic mRNA (*arsBHCrpaBslr0948*; ∼4000 pb) in response to arsenic treatment in WT (Figure 4B and Figure S6 in File S1). In the SARSR strain the *arsBHC* was expressed constitutively and as an operon with *rpaB* and *slr0948* (Figure 4B and Figure S8 in File S1). This data suggests that the intergenic region between *arsC* and *rpaB* lacks a transcriptional terminator strong enough to prevent read through from the *arsBHC* operon. The other gene under ArsR control was *sll0914*, which codes for a protein with a lipase/esterase domain. Expression of *sll0914* was analyzed by northern blot but no signal was detected in either WT or in SARSR. Therefore, *sll0914* expression was analyzed by qRT-PCR. *sll0914* was found to be expressed at low levels with similar Ct values to *arsI* using the same cDNA samples. Furthermore, its expression was induced by arsenite in the WT strain (Table S1) and constitutive (and higher than in the untreated WT strain) in the SARSR strain (Figure S8 in File S1). These results corroborated that *sll0914* is regulated by ArsR. To further clarify its role in arsenic resistance mutants in this gene were constructed in WT, SARSB and SARSR strains. All these mutant strains were as resistant as WT, SARSB or SARSR parental strains to arsenic (Figure S7 in File S1). Therefore, the role of Sll0914 in arsenic resistance is not clear, although its homology to lipases/esterases suggests that it could be involved in phospholipid degradation, releasing its phosphate component, which could protect against arsenate toxicity.

Analysis of the transcriptional profile of the SARSR strain after arsenite treatment indicated that SARSR strain did not induce a major reorganization of gene expression in response to arsenite as observed in the WT strain (Figures 1A and 4C). In fact, there were no genes differentially regulated between treated and untreated SARSR samples using our statistic test. This probably reflects that the high levels of expression of *arsBHC* operon in the SARSR strain allowed this strain to completely detoxify arsenite (exporting it outside the cells) in a short period of time and thus avoiding the stress response caused by arsenite. The fact that the SARSR strain did not show any stress response after arsenite treatment, lead us to re-evaluate its phenotype in response to arsenite. We have previously shown that SARSR and WT strains were similarly resistant to 1 mM arsenite in solid media but we did not check higher concentrations due to growth inhibition in our assay conditions. In order to test if the SARSR strain was resistant to arsenic, its growth in the presence of arsenate and arsenite was tested in liquid media (Figure 4D and E). The SARSR strain was more resistant to both arsenate and arsenite in the media than the WT strain. The SARSR strain was able to grow in the presence of 5 mM arsenite, while the WT strain was already affected by 3 mM arsenite and completely arrested at 5 mM arsenite (Figure 4D and Figure S4C in File S1). A similar behavior has been shown in *Shewanella* sp. ANA-3 *arsR2* and *arsR1* mutants which showed a reduced lag phase when inoculated in arsenite containing media [93]. In contrast, the SARSR strain presented a small, but consistent, delay in growth after inoculation in the presence of 100 mM of arsenate (Figure 4E and Figure S4D in File S1), although they reached higher final OD and chlorophyll content. These results suggest that over-expression of the *arsBHC* operon in the presence of high amounts of arsenate might be detrimental, but has a beneficial effect when arsenate concentration decreases (and probably arsenite accumulates in the media; Figure 4D and Figure S4D in File S1).

### Over expression of *arsB* confers metal sensitivity

**Figure 5:**
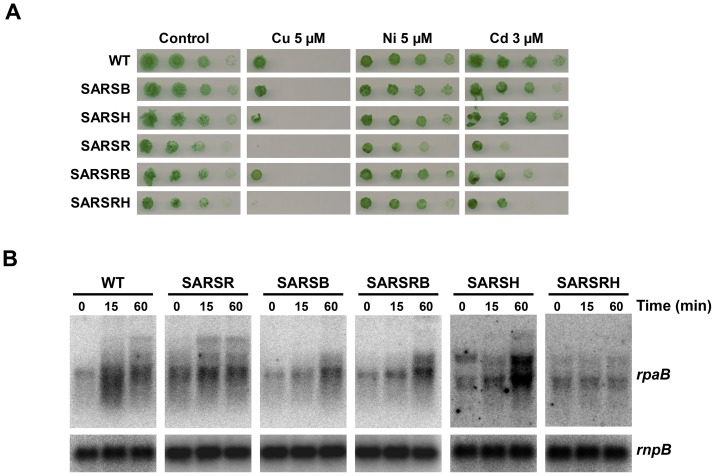
SARSR shows altered resistance to metals. A. Phenotypic characterization of mutants in arsenic resistance genes. Tolerance of the WT, SARSR, SARSB, SARSH, SARSRB and SARSRH strains to copper, nickel and cadmium wa^s^ examined. Tenfold serial dilutions of a 1 µg chlorophyll ml^−1^ cells suspension were spotted onto BG11C supplemented with the indicated metals concentrations. Plates were photographed after 5 days of growth. B. Northen blot analysis of *rpaB* expression in response to 1 mM arsenite treatment in WT, SARSR, SARSB, SARSH, SARSRB and SARSRH strains. Total RNA was isolated from cells grown in BG11C after addition of arsenite 1 mM. Samples were taken at the indicated times. The filters were hybridized with an *rpaB* probe and subsequently stripped and re-hybridized with an *rnpB* gene probe as a control.

The *arsBHC* operon has also been reported to be induced by the presence of other metals in the media in other microarray experiments [52]. Furthermore, *arsC* mutants have been described to be sensitive to Cd [52]. In order to clarify the role of the *arsBHC* operon in metal homeostasis the sensitivity of the mutants in these genes to the presence of different metals in the media was analyzed. While the SARSB and SARSH strains were as resistant as the WT to Ni, Cu and Cd, the SARSR strain was sensitive to the presence of the three metals (Figure 5A). The only genes that showed differential expression in the SARSR strain were *arsBHCrpaBslr0948*, suggesting that over-expression of any of these genes could be involved in this sensitivity. *arsB* codes for a Acr3 family of arsenite transporters and hence its over expression could alter membrane permeability to metals. Another possibility is that altered levels of other transcripts (*arsH*, *arsC* or *rpaB*) in the SARSR strain could affect metal sensitivity. In order to test these two possibilities we constructed double mutants in *arsR* and *arsB* (SARSRB) or *arsR* and *arsH* (SARSRH) and tested their sensitivity to metals in the media. *rpaB* expression levels were also checked in these strains. While double mutants in *arsH* and *arsR* (SARSRH strain) have a phenotype identical to single mutants in *arsR* respect to metal sensitivity, mutants in *arsB* and *arsR* (SARSRB strain) restored resistance to metals similar to single mutants in *arsB* (SARSB strain) and WT (Figure 5A). On the other hand, SARSB, SARSH, SARSRB and SARSRH strains presented similar levels of *rpaB* transcript levels that were lower than in the SARSR strain (Figure 5B). These results suggest that the metal sensitivity phenotype of the SARSR strain is mediated by *arsB* overexpression (that probably will include ArsB accumulation) and not by altered *rpaB* transcript levels. *arsR* mutation in *E. coli* is toxic due to over expression of *arsDBC* but experiments were carried out in LB media which is rich in metals and therefore the effect could be also mediated by specific metals. Even though *E. coli* ArsB and *Synechocystis* ArsB belong to non-related families of proteins, it is possible that high levels of expression of these membrane proteins, in *arsR* mutant strains, could impair membrane permeability.

## Conclusions

In summary we have shown that arsenate and arsenite produced similar genome-wide responses in *Synechocystis* sp. PCC 6803. These responses are dominated by a strong stress response, which included induction of the redox scavenging system and chaperones and repression of photosynthesis and growth related genes. This response is very similar in a mutant lacking the *arsB* gene, which is unable to detoxify arsenite, but is maintained for a longer time while is transient in the wild type strain. In contrast a mutant in all arsenate reductases (the SARS12 strain) showed a different response that included lower induction of metal resistance genes and Fe-S cluster biogenesis genes, suggesting that arsenite targets these processes in the WT strain. Moreover, the SARS12 strain expressed the *rpaB* gene at lower levels and presented higher expression levels of some of the RpaB repressed genes. Finally, the mutant strain lacking ArsR repressor (the SARSR strain) did not respond to arsenite, probably because it overexpressed ArsB, and was more resistant to arsenite than the WT strain. In contrast, the SARSR strain presented hypersensitivity to nickel, copper and cadmium which was lost in double mutants also lacking *arsB*. These suggested that overexpression of ArsB alters the membrane permeability to metals.

## Material and Methods

### Strains and growth conditions

*Synechocystis* strains used in this work are listed in Table S1. All these strains were grown photoautotrophically in BG11 medium [94] supplemented with 1 g L^−1^ NaHCO_3_ (BG11C) at 30°C under continuous illumination (50 µE m^−2^ s^−1^) and bubbled with a stream of 1% (v/v) CO_2_ in air. For plate cultures, medium were supplemented with 1% (w/v) agar. Kanamycin, chloramphenicol and spectinomycin were added to a final concentration of 50 µg mL^−1^, 20 µg mL^−1^ and 5 µg mL^−1^, respectively. In the BG11C low-phosphate medium, the concentration of K_2_HPO_4_ was reduced to 30 µM. 1 mM of NaAsO_2_ or 50 mM of Na_2_HAsO_4_, 5 µM NiSO_4_, 5 µM CuSO_4_, or 3 µM CdSO_4_ were added when required.

All experiments were performed using cultures from the mid-logarithmic phase (3 to 5 µg chlorophyll ml^−1^). *Synechocystis* strains and their relevant genotypes are described in Table 1. *E. coli* DH5α cells were grown in Luria broth medium and supplemented with 100 µg ml^−1^ ampicillin, 50 µg ml^−1^ kanamycin, 20 µg ml^−1^ chloramphenicol and 100 µg ml^−1^ spectinomycin when required.

**Table 1:** *Synechocystis* strains used in this work.

*Synechocystis* strains	Genotype	Mutated ORF(s)	Source or Study
WT	*Synechocystis* sp. PCC 6803		Lab collection
SARS12	*arsC::CK1 arsI1::CC1* Δ*arsI2::ΩSp*	*slr0946, slr6037, sll5104*	[23]
SARSR	*arsR::CC1*	*sll1957*	[24]
SARSB	*arsB::CK1*	*slr0944*	[24]
SARSH	*arsH::CK1*	*slr0945*	[24]
SARSRB	*arsR::CC1 arsB::Kmr*	*sll1957, slr0944*	This study
SARSRH	*arsR::CC1 arsH::CK1*	*sll1957, slr0945*	This study
sll0914	*sll0914::ΩSp*	*sll0914*	This study
SARSR sll0914	*arsR::CC1 sll0914::ΩSp*	*sll1957, sll0914*	This study
SARSB sll0914	*arsB::CK1 sll0914::ΩSp*	*slr0944, sll0914*	This study

### Insertional mutagenesis of *Synechocystis* genes

For the *sll0914* insertional mutant, a 1093 bp PCR fragment was amplified from total genomic DNA using two-step PCR method, synthesizing overlapping fragments that incorporate a *BamH*I restriction site, and was cloned into pGEMT to generate pSll0914.1. Then, an Sm/Sp cassette was cloned into *BamH*I site generating pSll0914.2 plasmid. This plasmid was used to transform WT, SARSR and SARSB strains. The pARSH2(+) and pARSB2(+) targeting plasmids [24] were used to transform a SARSR strain obtaining the SARSRH and SARSRB mutants. Correct integration and segregation of all mutants were confirmed by PCR analysis. All the oligonucleotides used for cloning and insertional mutagenesis are described in Table S6.

### RNA isolation, Northern blot analysis, microarray hibridization and data analysis

Total RNA was isolated from 30 ml samples of *Synechocystis* cultures at the mid-exponential growth phase (3 to 5 µg chlorophyll ml^−1^). Extractions were performed by vortexing cells in the presence of phenol-chloroform and acid-washed baked glass beads (0.25–0.3 mm diameter) as previously described [95].

For Northern blotting, 10 ug of total RNA was loaded per lane and electrophoresed on denaturing formaldehyde-containing 1% agarose gel. Transfer to nylon membranes (Hybond N-plus, GE Healthcare), pre-hybridization, hybridization and washes were performed as recommended by the manufacture. Probes for Northern blot hybridization were synthesized by PCR using oligonucleotide pairs: arsC_F–arsC_R, rpaB_F–rpaB_R, and slr0948_F–slr0948_R, trxA_1-trxA_2, trxB1-trxB2, trxQ_F-trxQ_R, prxII_F-prxII_R, 1cys-Prx_F-1cys-Prx_R, 2cys-Prx_F-2cys-Prx_R, prxQ1_F-prxQ1_R, prxQ2_F-prxQ2_R (see Table S6) for *arsC*, *rpaB, slr0948, trxA, trxB, trxQ, prxII, 1cysprx, 2cysprx, prxQ1* and *prxQ2*, respectively. As a control, in all cases the filters were stripped and re-probed with a 580-bp *Hind*III-*Bam*HI probe from plasmid pAV1100 containing the constitutively expressed RNase P RNA gene (*rnpB*) from *Synechocystis* sp. strain PCC 6803 (Vioque, 1992). DNA probes were ^32^P labeled with a random-primer kit (Amersham Biosciences) using [α-^32^P]dCTP (3,000 Ci/mmol). Hybridization signals were quantified with a Cyclone Phosphor System (Packard).

For microarray analysis 0.2 µg of RNA were transformed to cRNA using Low Input Quick Amp WT Labeling Kit from Agilent. cRNA was labeled with Cy3 and labeled cRNA was applied to 8X15K arrays. Signal intensities for probes were obtained from the scanned microarray image using Agilent Technologies' Feature Extraction software and quantile normalized. Differentially expressed genes were selected using Limma implemented in One Channel GUI with a *p*<0.05 and at least 2.5 fold change. Gene groups differentially expressed in different genotypes were identified using GSEA tool [96] using hand-compiled gene lists (Table S7) that include functional categories from cyanobase, GO annotation and literature curated gene list (see supplementary material). The data discussed in this publication have been deposited in NCBI's Gene Expression Omnibus and are accessible through GEO Series accession number GSE51383 (http://www.ncbi.nlm.nih.gov/geo/query/acc.cgi?acc=GSE51383).

### RT-PCR and qRT-PCR

cDNA were prepared with Quantitech reverse transcriptase kit from Qiagene using 1 µg RNA treated with TurboDNAse (Ambion) and following manufacturer's instructions. 2 µl of cDNA were used for each PCR reaction. For qRT-PCR, cDNA was diluted ten-fold and 5 µl were used for each Real-time PCR reaction which were performed using SensiFAST SYBR Hi-ROX Kit (BioLine), and the signals were detected on an ABI StepONE real time PCR instrument (Applied Biosystems) following manufacturer's instructions. The expression levels of the genes of interest were normalized to the constitutive *rnpB* gene. The fold change was calculated using the ΔΔC^t^ method. The results shown are from three independent RNA samples. Primers used for the analysis are indicated in Table S6 and are identified with Q as the first letter in their names.

## Supporting Information

File S1 **Supporting Figures.**
(PDF)

Table S1 **Genes differentially expressed after arsenic treatments in WT Synechocystis cells.**
(XLSX)

Table S2 **Gene list enriched in WT treated with 1 mM arsenite or 50 mM arsenate.**
(XLSX)

Table S3 **Genes differentially expressed between SARS12 and WT.**
(XLSX)

Table S4 **Gene list enriched in SARS12 vs WT treated with 50 mM arsenate.**
(XLSX)

Table S5 **Genes differentially regulated in untreated cells of SARSR when compared to WT.**
(XLSX)

Table S6 **Oligonucleotides used in this work.**
(DOCX)

Table S7 **Hand curated gene list used in the GSEA analysis.**
(XLSX)
