# Supplementary material for: Genomic Responses to Arsenic in the Cyanobacterium Synechocystis sp. PCC 6803
Source: PLoS One. 2014 May 5;9(5):e96826. doi: 10.1371/journal.pone.0096826 (PMC4010505; doi:10.1371/journal.pone.0096826)
Supplement: File S1 — Supporting Figures. (PDF) [file pone.0096826.s001.pdf]

Figure S1.

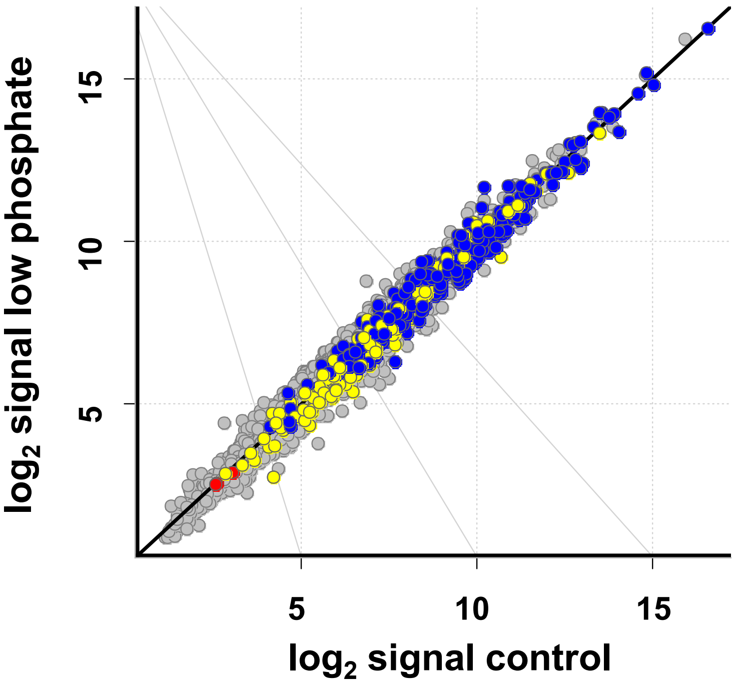

Figure S1. Low phosphate media does not alter gene expression in the wild type. Scatter plot showing $\log_2$ of signal obtained from all genes in WT cells grown in BG11C (x-axis) and low phosphate BG11C (y-axis). CTR up-regulated genes are colored in yellow, CTR down-regulated genes in blue and the *arsBHC* operon in red.

Figure S2.

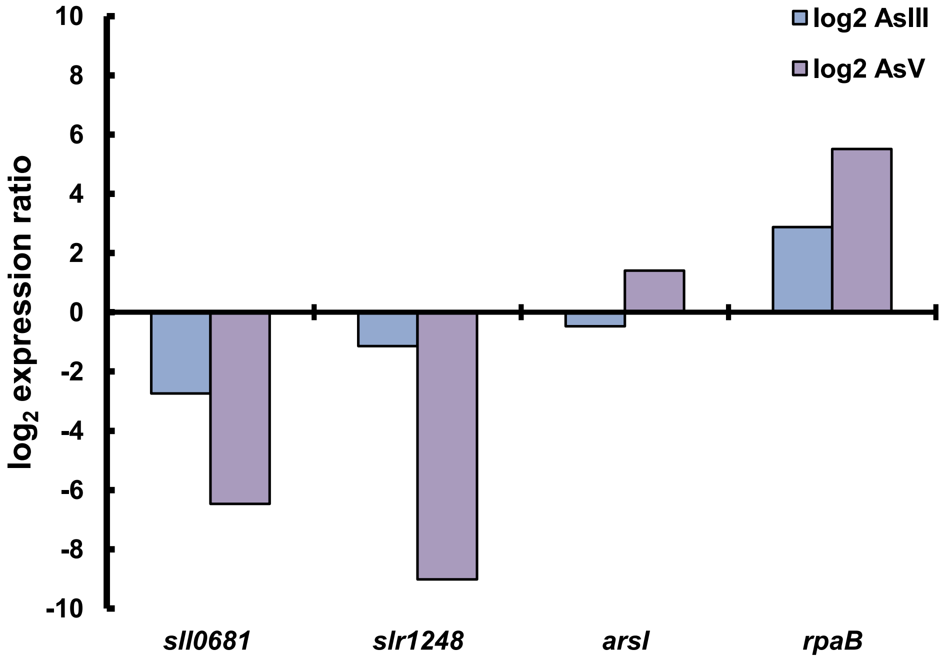

Figure S2. Expression of selected genes in response to arsenate and arsenite in WT cells analyzed by qRT-PCR.

Analysis of *sll0681* (pst-1), *slr1248* (pst-2), *arsI* (*sll5104* and *slr6037*) and *rpaB (slr0947)* expression after 1h of 1 mM arsenite (blue bars) or 50 mM arsenate (purple bars) in WT cells. Data are the mean of two biological repeats which are independent experiments from the samples used for microarrays.

Figure S3

A

B

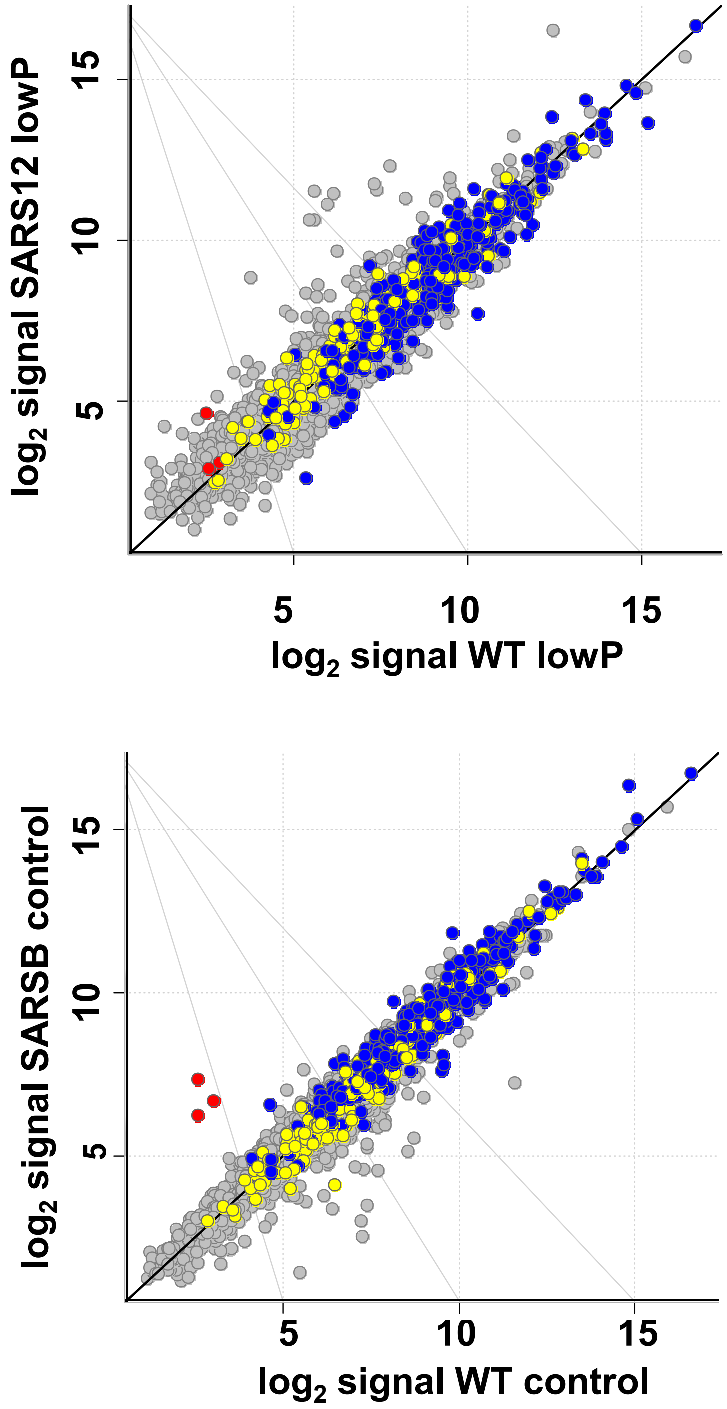

Figure S3. SARS12 and SARSB have similar gene expression profiles to WT in untreated conditions.

A. Scatter plot showing $\log_2$ of signal obtained from all genes in samples from WT grown in low phosphate BG11C (x-axis) and SARS12 grown in low phosphate BG11C (y-axis). CTR up-regulated genes are colored in yellow, CTR down-regulated genes in blue and the *arsBHC* operon in red.

B. Scatter plot showing $\log_2$ of signal obtained from all genes in samples from WT grown in BG11C (x-axis) and SARSB grown in BG11C (y-axis). CTR up-regulated genes are colored in yellow, CTR down-regulated genes in blue and the *arsBHC* operon in red.

Figure S4.

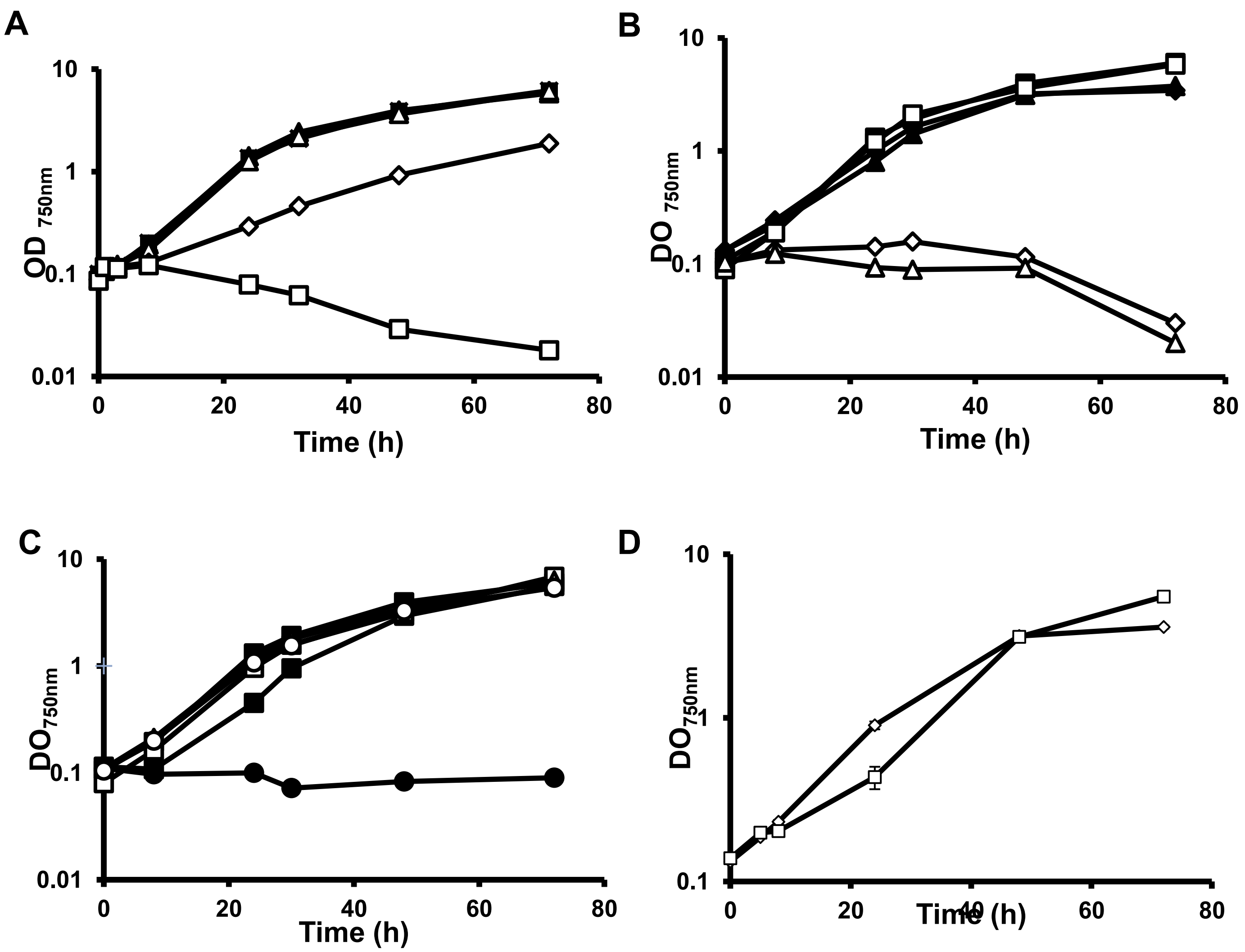

Figure S4. Growth of SARSB, SARS12 and SARSR in the presence of arsenic.

A. Growth of WT and SARSB strains in different arsenite concentrations. Exponentially growing cells of WT (filled symbols) and SARSB (open symbols) were diluted to 0.5 µg chl ml^{-1} (equivalent to 0.1 OD_{750nm}) in media containing 100 µM arsenite (squares), 50 µM arsenite (diamonds) or without added arsenite (triangles). Growth was monitored following OD_{750nm}.

B. Growth of WT and SARS12 strains in the presence of different arsenate concentrations. Exponentially growing cells of WT (filled symbols) and SARS12 (open symbols) were diluted to 0.5 µg chl ml^{-1} (equivalent to 0.1 OD_{750nm}) in low phosphate media containing 100 mM arsenate (triangles), 50 mM arsenate (diamonds) or without added arsenate (squares). Growth was monitored following OD_{750nm}.

C. Growth of the WT and SARSR strains in the presence of different arsenite concentrations. Exponentially growing cells of WT (filled symbols) and SARSR (open symbols) were diluted to 0.5 µg chl ml^{-1} (equivalent to 0.1 OD_{750nm}) in BG11C containing 3 mM arsenite (triangles), 5 mM of arsenite (circles) or without added arsenate (squares). Growth was monitored following OD_{750nm}.

D. Growth of the WT and SARSR strains in the presence of arsenate. Exponentially growing cells of the WT (diamonds) and SARSR (squares) strains were diluted to 0.5 µg chl ml^{-1} (equivalent to 0.1 OD_{750nm}) in low phosphate media containing 100 mM of arsenate. Growth was monitored following OD_{750nm}; data represent average of 3 independent experiments and error bars represent SE.

Figure S5

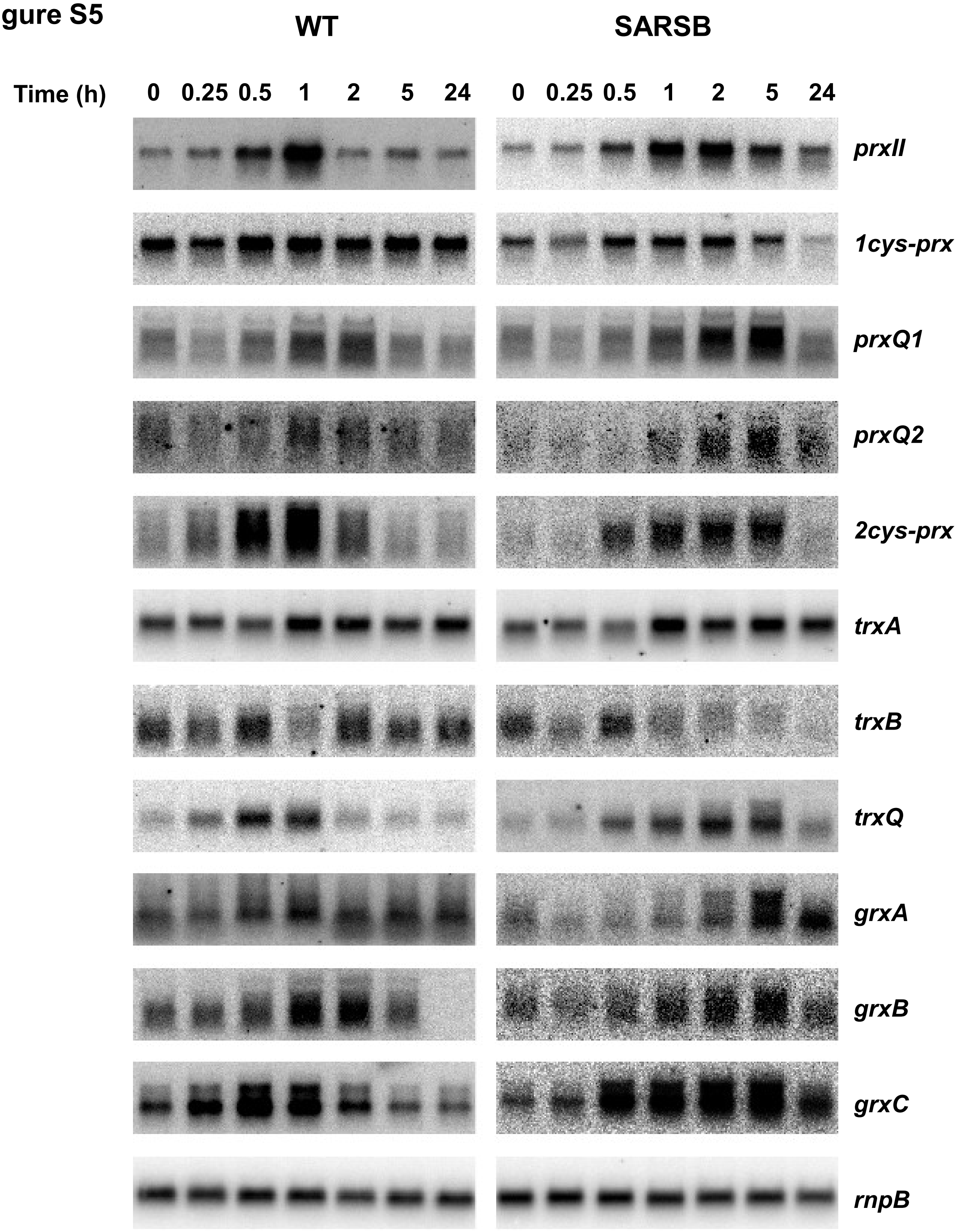

Figure S5. Expression of redox genes in response to 1 mM arsenite in the WT and SARSB strains analyzed by Northern blot.
Northern blot analysis of the expression of *prxII*, *1cys-prx, prxQ1, prxQ2, 2cys-prx, trxA, trxB, trxQ, grxA, grxB and grxC* after 1 mM arsenite addition. Total RNA was isolated from WT and SARSB cells grown in BG11C after addition of arsenite 1 mM. Samples were taken at the indicated times. The filters were subsequently hybridized with *prxII*, *1cys-prx, prxQ1, prxQ2, 2cys-prx, trxA, trxB, trxQ, grxA, grxB, grxC* and *rnpB* gene probes.

Figure S6.

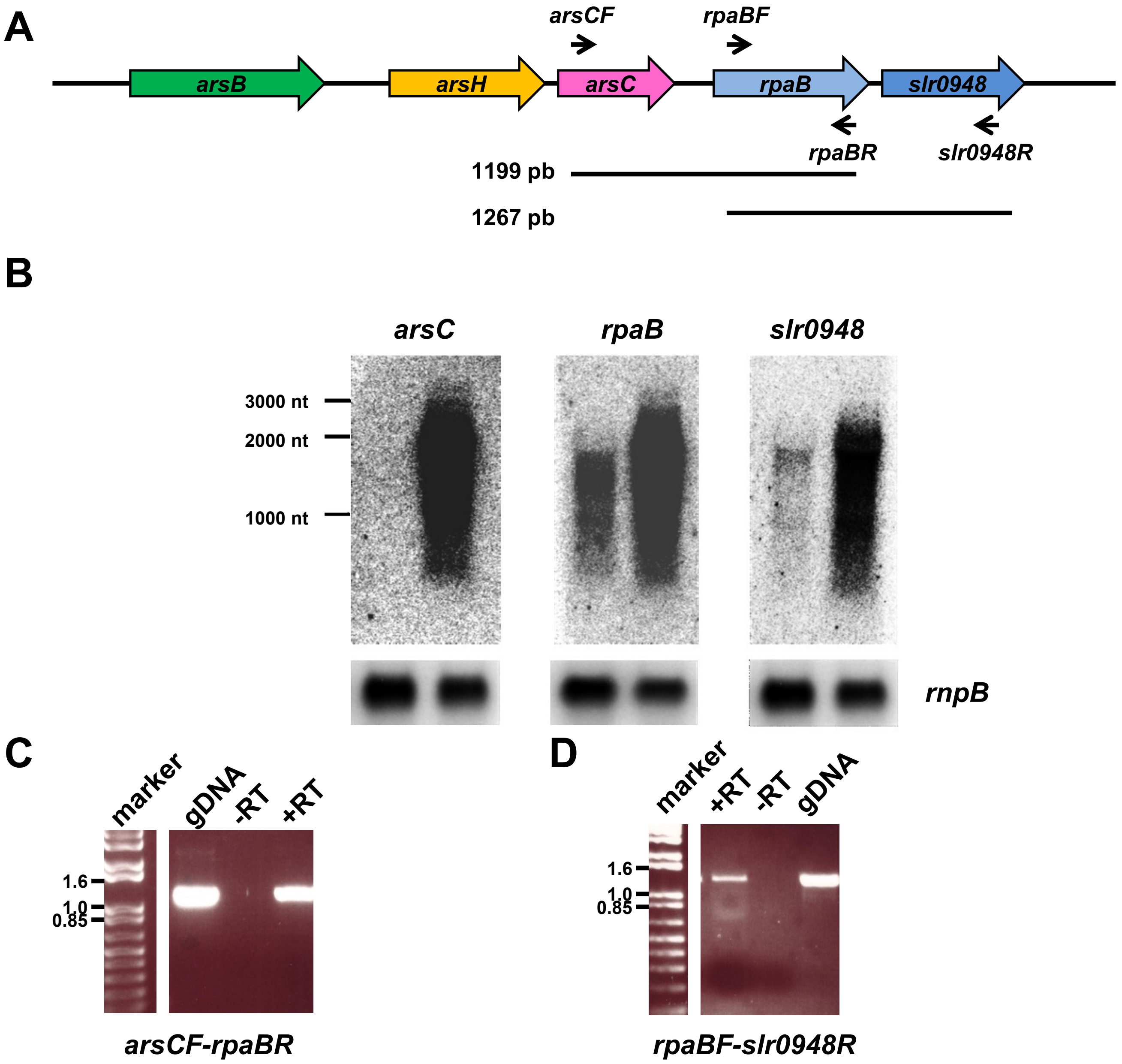

Figure S6. *arsBHCrpaBslr0948* are expressed as an operon in response to arsenic.

A. Schematic representation of the *arsBHC-rpaB* genomic region.
B. Northern blot analysis of the *arsBHC-rpaBslr0948* operon. RNA was isolated from WT cells grown in BG11C before or 1h after the addition of 1 mM arsenite. The filters were hybridized with *arsC*, *rpaB* and *slr0948* probes and subsequently stripped and re-hybridized with an *rnpB* gene probe as a loading control. A RNA ladder (ssRNA Ladder NEB catalog # N0362S) was also loaded to compare the molecular sizes.
C. RT-PCR analysis of the *arsBHC-rpaB* operon. cDNA was generated using RNA extracted from WT cells grown in BG11C 1h after the addition of 1 mM arsenite. 10 µl of the PCR reaction using oligolucleotides arsCF-rpaBR, was loaded in a 1% agarose gel. (+) RT treatment (-) without RT treatment. A DNA ladder (1kb plus, Invitrogen catalog # 10787-018) was also loaded to compare the molecular sizes.
D. RT-PCR analysis of the *rpaB-slr0948* operon. cDNA was generated using RNA extracted from WT cells grown in BG11C 1h after the addition of 1 mM arsenite. 10 µl of a PCR reaction, using oligolucleotides rpaBF-slr0948R, was loaded in a 1% agarose gel. (+) RT treatment (-) without RT treatment. A DNA ladder (1kb plus, Invitrogen catalog # 10787-018) was also loaded to compare the molecular sizes.

Figure S7.

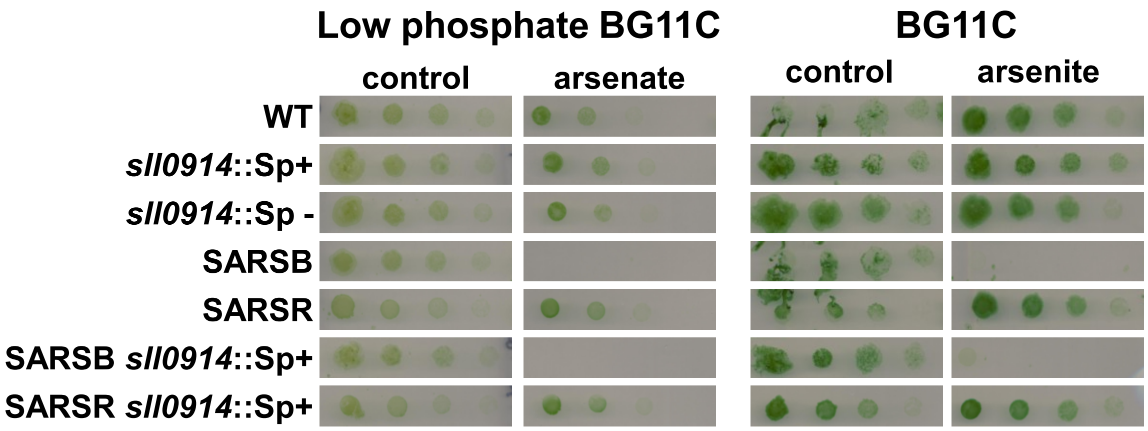

Figure S7. *sll0914* mutation does not affect arsenic resistance.

Phenotypic characterization of mutants in arsenic resistance genes. Tolerance of WT, sll0914::Sp+, sll0914::Sp-, SARSB, SARSR, SARSBsll0914::Sp+ and SARSR sll0914::Sp+ strains to 50 mM arsenate (left panel) and 1 mM arsenite (rigth panel) was examined. Ten-fold serial dilutions of a 1 µg chlorophyll ml^{-1} cells suspension were spotted onto BG11C supplemented with the indicated metal concentrations. Plates were photographed after 5 days of growth.

Figure S8.

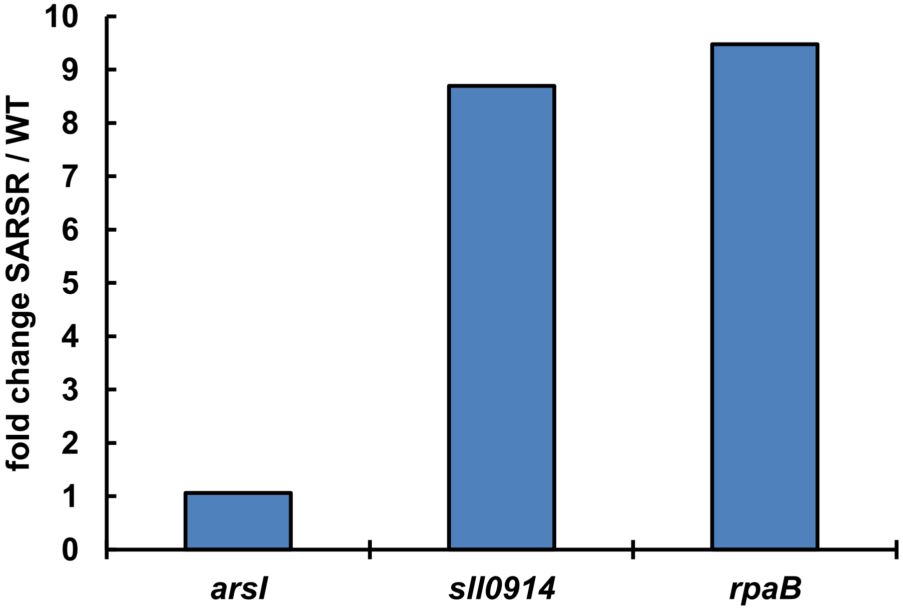

Figure S8. Expression of *arsI*, *sll0914* and *rpaB* genes in the SARSR strain analyzed by qRT-PCR.

Analysis of *arsI* (*sll5104* and *slr6037*), *sll0914* and *rpaB (slr0947)* expression in control conditions. Bars represent ratio between SARSR vs. WT samples. Data are the mean of two biological repeats which are independent experiments from the samples used for microarrays.
