# Supplementary material for: Genomic Responses to Arsenic in the Cyanobacterium Synechocystis sp. PCC 6803
Source: PLoS One. 2014 May 5;9(5):e96826. doi: 10.1371/journal.pone.0096826 (PMC4010505; doi:10.1371/journal.pone.0096826)
Supplement: Table S6 — Oligonucleotides used in this work. (DOCX) [file pone.0096826.s007.docx]

Table S6. Oligonucleotides used in this work

| Oligonucleotide | Sequence 5’ to 3’ |
| --- | --- |
| arsC_F | CTGTCGCTCCCAAATGGCAG |
| arsC_R | CGATCAGGTTTTCCACCCGTTC |
| rpaB_F | CATGATTGGCTATGAAGTG |
| rpaB_R | CAGAATGCGTTGGAACAGG |
| slr0948_F | CAATCGTATTGTTCGTTTA |
| slr0948_R | GGAATATTGGCTCCTAGG |
| sll0914_F1 | ATGCCCGCCCCCAAAATTATT |
| sll0914_R1 | GGATTAACGGATCCCACAAAG |
| sll0914_F2 | CGGACTTTGTGGGATCCGTTA |
| sll0914_R2 | CCAAAATGCACTCAGTCAAAT |
| slr1248_R | GGCGATGAAGGAACTAACCA |
| slr1248_L | TGGGAATTCATCGTCAGTCA |
| sll0681_L | CCGTCAGTATTGGGCTGATT |
| sll0681_R | GGCTGGTAACAGGATTCCAA |
| grxA250F | GGCTGTCTCGGCAAAAATTG |
| grxA250R | GGTCCAACTTGCCTGCACCATC |
| grxB331F | GGCTAATTTGTTCAACTGGC |
| grxB331R | CTAGGCTGGGTTAGGAGGAG |
| grxC295F | GCAAGAATTGATCAGTTGGTC |
| grxC295R | GCCACTTCTAACATTTCCTGC |
| trxA_1 | TTCCAGTATGAGTGCTACCC’ |
| trxA_2 | AAGCCAGCGCTTAAAGATAT |
| trxB1 | GACCATTCATATGAGTTTAC |
| trxB2 | CAAATAGCTCGAGTCAGTTTAGGG |
| trxQ_F | ACTGGATTAATATCTTTGCG |
| trxQ_R | CTAAAGGTTTGTTGTCG |
| prxII_F | GAGGTACATATGACCCCCGAACG |
| prxII_R | GGTTTCACTCGAGTAATCAC |
| 1cys Prx_F | GCTACATATGGCCTTACAACTCGGTGATG |
| 1cys Prx_R | GCTACTCGAGCTTATTGGGTTGGGGGGTC |
| 2cys Prx_F | GCTACATATGACAGAGGTATTAAGGGTAG |
| 2cys Prx_R | GCTACTCGAGCTAAGGTTCCGCCACTGTCTC |
| prxQ1_F | GAATTTTTTTCATATGGCCACTGCCTTAG |
| prxQ1_R | CCAAGGCTCGAGGATTTGCTTGC |
| prxQ2_F | CCTGGGGCATATGCAACCAGAGTTG |
| prxQ2_R | CGGGATTATTTACGTCGACGGGGAG |
| Q_arsI_F | TGGTAAACAGGCCATTGTC |
| Q_arsI_R | CAGAAGGCCAATGATCCAAC |
| Q_rnpB_F | CACAGAAAAATACCGCCCT |
| Q_rnpB_R | TTAGTCAGCAGAAAAACCC |
| Q_rpaB_F | ATCCGACGCATACTGGAAAC |
| Q_rpaB_R | GCATCATCACATCCAACACC |
| Q_sll0914_F | AAGCCTTCCCTGGTGAGAAC |
| Q_sll0914_R | GGGCGGAATCAAAATTGTAC |
